# Intramedullary nailing and plating osteosynthesis in the correction of post-traumatic deformity in late-diagnosed distal radius fractures: a retrospective comparison study

**DOI:** 10.1186/s12891-019-2605-1

**Published:** 2019-05-21

**Authors:** Alvin Chao-Yu Chen, Chun-Ying Cheng, Chun-Jui Weng, Ying-Chao Chou

**Affiliations:** 1 Department of Orthopaedic Surgery, Bone and Joint Research Center, Chang Gung Memorial Hospital–Linkou, 5th, Fu-Shin Street, Kweishan District, Taoyuan, 333 Taiwan, Republic of China; 2 Chang Gung University College of Medicine, Taoyuan City, Taiwan

**Keywords:** Deformity, Distal radius fracture, Malunion, Osteotomy, Intramedullary nail, Locking plate

## Abstract

**Background:**

Various surgical modalities are available for correction of deformity in late-diagnosed distal radius fractures. This study compared surgical outcomes between intramedullary nailing and plating osteosynthesis in open-wedge osteotomy.

**Methods:**

We retrospectively reviewed 47 unilateral distal radius fractures that underwent open-wedge osteotomy at more than 4 weeks after injury between 2006 and 2011. A locally resected exuberant callus was used as the bone graft. Two types of fixation were used: intramedullary nail in 22 patients (group A) and locking plate in 25 patients (group B). Radiographic analysis including radial height, radial inclination, and volar tilt were performed preoperatively and 2-year postoperatively. The Modified Mayo Wrist Score (MMWS) was used for functional evaluation and a 10-point visual analog scale (VAS) for residual pain assessment. Patient satisfaction was self-reported as a 5-point scale. Radiographic data, functional outcomes, and surgical complications were compared between the two groups.

**Results:**

All fractures achieved bone union without major complications. The MMWS averaged 84.8 ± 9.7 in group A and 85.2 ± 8.4 in group B, without significant differences (*p =* 0.436). Instead, significant differences were found in mean wrist flexion (73.6 ± 7.9 vs. 69.6 ± 7.8 degrees; *p =* 0.042), patient satisfaction (3.6 ± 1.1 vs. 2.9 ± 1.2; *p =* 0.034), postoperative radial height (11.6 ± 2.6 vs. 10.2 ± 3 mm; *p =* 0.039) and inclination (20.8 ± 2.8 vs. 17.7 ± 4.1 degrees; *p =* 0.004), and implant-related complications (9% vs. 36%; *p =* 0.03). There were no significant differences in other assessment items including postoperative grip strength, pain scale, supination/pronation/extension, volar tilt, correction angles of all three parameters, and general complication rate. Four patients in group A (18%) and 2 in group B (8%) experienced postoperative paresthesia of the surgical hand; no significant difference was noted. All except one patient in group B had full recovery of neurological symptoms.

**Conclusions:**

Open-wedge osteotomy with either an intramedullary nail or locking plate fixation yielded encouraging radiographic and functional outcomes. Intramedullary nail fixation may facilitate restoration of radial height and inclination with better wrist flexion, less implant-related complications, and greater patient satisfaction.

## Background

Given the recent advances in the surgical intervention of distal radius fractures [1, 2], posttraumatic deformity that may lead to alternation of biomechanics of the wrist and function impairment in the hand and forearm [3–5] is still common sequelae in late-diagnosed fractures [6–8]. Loss of the normal volar tilt in the sagittal plane, decreased radial inclination in the frontal plane, and loss of radial height have been reported to be common deformities following extra-articular fractures [8]. Numerous surgical modalities in treatment of acute fractures of the distal radius have been proposed. Among these, intramedullary nails and locking plates are currently available devices for osteosynthesis after reduction and fixation [9–11]. Both systems adopt a locking mechanism, in which a thread of the screw locks into the threaded hole in the intramedullary nail or the plate to create a fixed-angle construct for securing the distal metaphysis fragment. The aim of this retrospective study was to describe our experience using open osteotomy, local bone grafting, and fracture stabilization with two different devices for treatment of posttraumatic deformity in late-diagnosed fractures of the distal radius. The results were compared for intramedullary nailing and plating osteosynthesis based on clinical outcomes, radiographic analysis, and surgical complications.

## Methods

### Patient data

Ethical committee approval was obtained from Chang Gung Institutional Review Board (IRB 201801094B0). A total of 631 patients were identified in our surgical database that underwent surgical reduction and internal fixation for unilateral distal radius fracture between 2006 and 2014. This study recruited extra-articular fractures that underwent internal fixation 1 month or more after injury. Patients with open fractures and multiple trauma were excluded. Overall, 47 fractures in 47 patients with completion of at least 2 years follow-up were enrolled and divided into 2 groups based on the types of internal fixation. There were 2 types of implants including intramedullary nail in 22 patients (group A) and locking plate in 25 patients (group B). In group A, there were 14 females (68%) and 8 males (32%) with an average age of 59.1 ± 10.9 years (range 32–81). Left-side fractures were present in 13 patients (59%) and right-side fractures in 9 patients (41%); 10 patients (45%) had lesions in the dominant hand. Comminuted fractures were identified in 8 patients (36%). Time from injury to the index surgery averaged 11.1 ± 4.7 weeks (range 4–18). In group B, there were 11 females (44%) and 14 males (56%) with an average age of 50.4 ± 17.8 years (range 20–78). Left-side fractures occurred in 12 patients (48%) and right-side fractures in 13 patients (52%); 14 patients (52%) had fractures in the dominant hand. Comminuted fractures were identified in 10 patients (40%). Time from injury to the index surgery averaged 12.0 ± 6.6 weeks (range 4–28). Demographic data are compared in Table 1. Written informed consents were obtained from all the patients.

**Table 1:** Demographic data of patients

Characteristics	Group A^a^ (*N* = 22)	Group B^b^ (*N* = 25)	*P-value* ^*^
Mean age (years)	56.8 ± 12.4	50.4 ± 17.8	0.058
Sex			0.178
Women	14 (68%)	11 (44%)	
Men	8 (32%)	14 (56%)	
Injured wrist			0.447
Left	13 (59%)	12 (48%)	
Right	9 (41%)	13 (52%)	
Dominant side injury	10 (45%)	13 (52%)	0.654
Fracture comminution^c^	8 (36%)	10 (40%)	0.798
Time to surgery (weeks)	11.1 ± 4.7	12.0 ± 6.6	0.280

^*^P-values < 0.05 are considered statistically significant

^a^Patients who underwent intramedullary nailing

^b^Patients who underwent plating osteosynthesis

^c^Comminution of metaphyseal area

### Surgical procedure

#### Intramedullary nailing

**Fig. 1:**
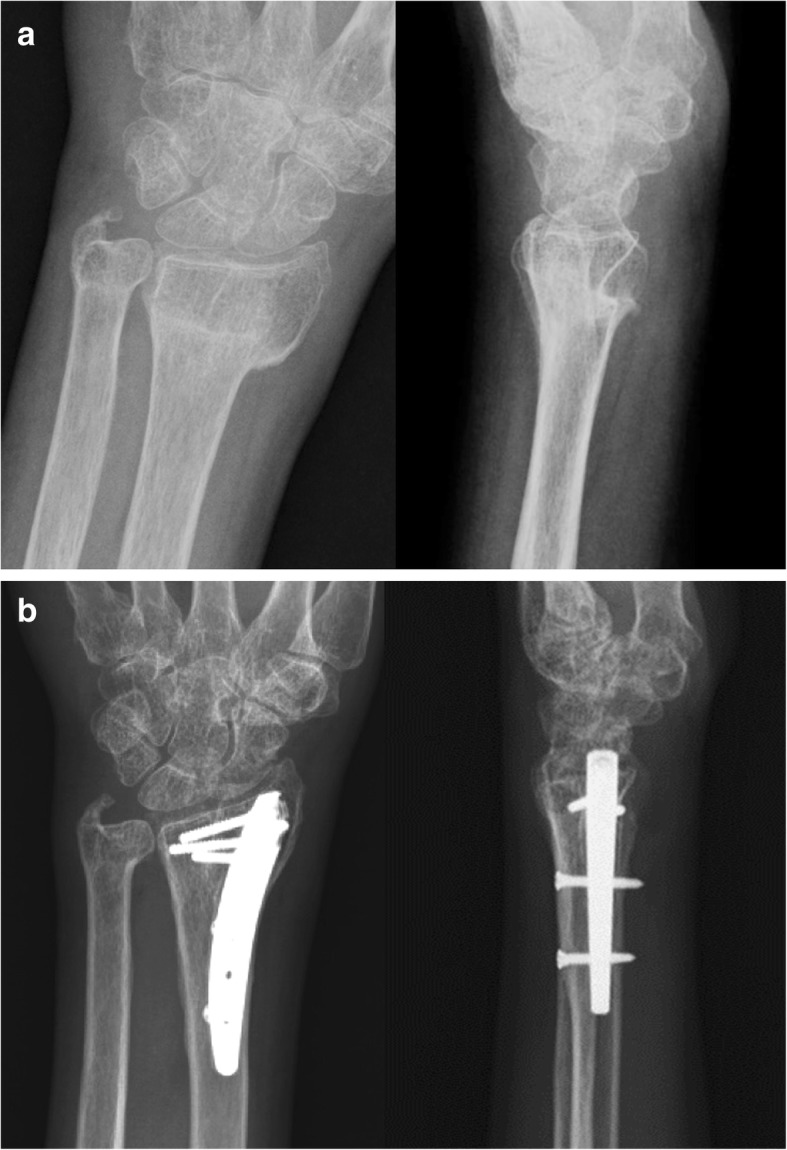
A 63-year-old female patient with distal radius fracture of 16 weeks. **a** Preoperative radiographs showing malunion, posteroanterior, and lateral projections. **b** Postoperative radiographs with intramedullary nail 2 years after surgery follow-up showing posteroanterior and lateral projections

Surgical exploration for fracture deformity began with a 2-cm longitudinal incision ulnar to Lister’s tubercle on the dorsal wrist. Osteotomy was achieved through the malunion site using a 5-mm osteotome and transfixed temporally with Kirschner wires with manual reduction under C-arm fluoroscopic assistance. Another 1-cm incision was made over the radial styloid with blunt soft tissue dissection and meticulous protection of superficial radial sensory nerve. Gentle dissection through the interval of the first and second dorsal extensor compartments was achieved using a starter awl to create a cortical bone window. This was followed by tapping and sequential broaching into the intramedullary canal until a proper fit was achieved. After sizing and trialing, a MICRONAIL® (Wright Medical Technologies, Arlington, TN) of the measured size was gently inserted through the pre-taped track into the medullary canal of the distal radius. Three distal fixed-angle locking screws and two proximal interlocking screws were then applied through the guiding system. After satisfactory realignment and secure fixation were confirmed by fluoroscopy, all provisionally transfixed Kirschner wires were removed and the guide system was dissembled from the intramedullary nail (Fig. 1). A locally resected exuberant callus from the nascent malunion was morselized to serve as a bone graft for the osteotomy site. The wound was then closed layer by layer.

#### Plating osteosynthesis

**Fig. 2:**
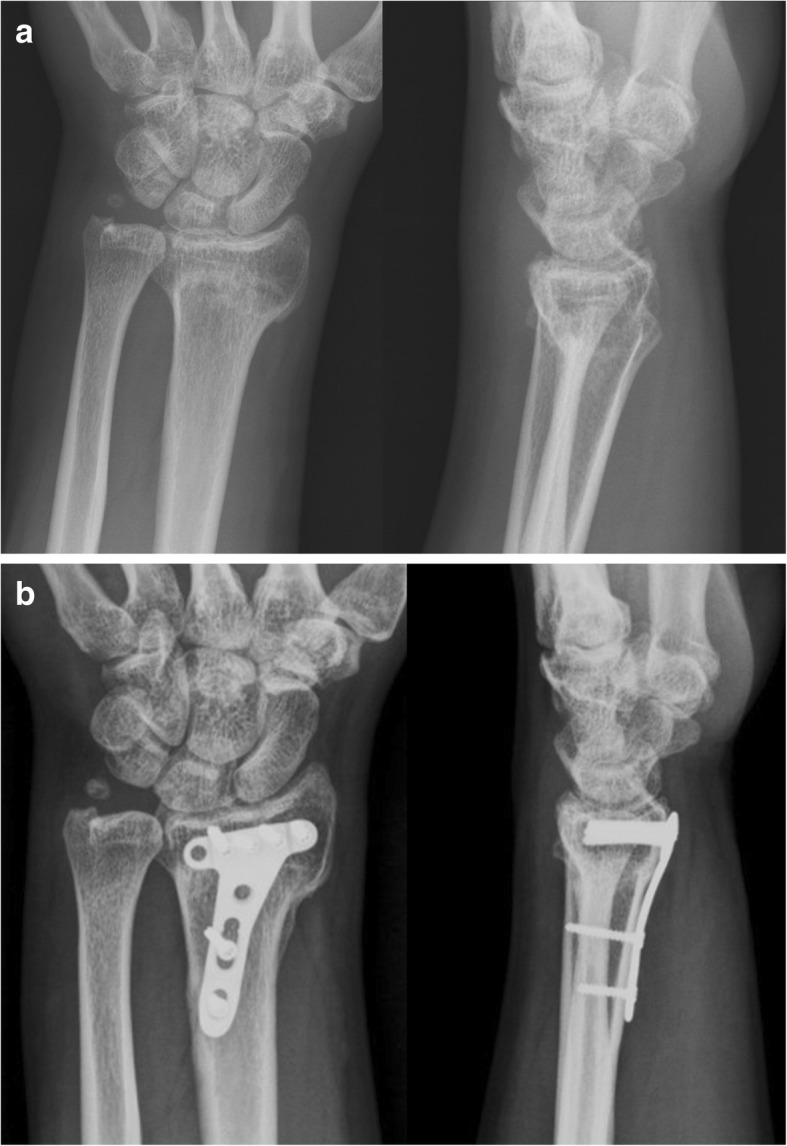
A 39-year-old male patient with a distal radius fracture of 28 weeks. **a** Preoperative radiographs showing malunion, posteroanterior, and lateral projections. **b** Postoperative radiographs of volar locking plate 2 years after surgery showing posteroanterior and lateral projections

A Henry approach was applied over the volar wrist. The flexor carpi radialis tendon sheath was opened with the flexor carpi radialis and the digital flexor were retracted ulnarly. The pronator quadratus was incised from its radial aspect and elevated to explore the fracture site. Osteotomy was performed through the malunion site using a 5-mm osteotome followed by manual reduction and provisional fixation with Kirschner wires under C-arm fluoroscopic assistance. A 2.4-mm titanium juxta-articular volar plate (Synthes Ltd., Paoli, PA, USA) was applied and temporarily fixed with a single compression screw in the proximal gliding hole. After confirmation of sufficient reduction and optimal plate location, the distal locking screws were then positioned. The proximal locking screws were inserted next. Then all the Kirschner wires were removed (Fig. 2). Locally resected exuberant callus was morselized and used to fill the osteotomy gap. The pronator quadratus was then reattached and wound was approximated with subcutical suture.

### Functional and radiographic survey

Functional assessment included range of motion of the wrist and forearm, grip strength, residual wrist pain, and satisfaction [12]. The Modified Mayo Wrist Score (MMWS) was used for a total count of 100 points and graded into 4 categories [13]. Pain score was rated using a 10-point visual analog scale (VAS). Patient satisfaction was self-reported in a 5-point scale that ranged from 1 very dissatisfied to 5 very satisfied. Treatment outcome was compared between the 2 groups at 2-years postoperatively. The osseous union was documented according to both a clinical and radiographic assessment. Nonunion, malunion, infection, or complications associated with the implant, such as pain or tenosynovitis, were also based on the review of medical records. Radiographic analyses including standard postero-anterior and lateral projections were performed on postoperative X-ray taken the next day and after 2-year follow-up. Three radiographic parameters: radial height, radial inclination, and volar tilt were evaluated.

### Statistical analysis

Descriptive statistics were calculated for key variables [14]. For normally distributed data (patient age and time from index to surgery), an independent sample t-test was used. For data that were not normally distributed (MMWS), the Mann-Whitney rank sum test was used. For categorical data (sex, injured side, injured hand dominance, fracture comminution, and complication rate), a chi-square test was used. A *p*-value of < 0.05 was considered statistically significant.

## Results

### Clinical outcomes

Clinic follow-up averaged 27.7 ± 4.7 months (range, 24 to 38) in the nail group; 28.8 ± 4.8 months (range, 24 to 38). The osseous union was confirmed both clinically and radiographically at 3-months after surgery in all 47 patients. Functional outcomes were measured and compared for the two groups 2 years after surgery and included the MMWS, grip strength, range of motion, pain scores, and patient satisfaction (Table 2). The MMWS, grip strength, pain score and range of motion including supination, pronation and extension showed no statistical differences between the two groups. Only flexion and patient satisfaction showed significant differences between groups A and B.

**Table 2:** Functional outcome

Items	Group A^a^ (*N* = 22)	Group B^b^ (*N* = 25)	*P-value* ^*^
MMWS^c^	84.8 ± 9.7	85.2 ± 8.4	0.436
Grip strength (kg)	25.6 ± 4.6	26.6 ± 4.1	0.228
Range of motion (degrees)			
Supination	77.3 ± 8. 1	78.4 ± 6.7	0.379
Pronation	75.9 ± 7.2	76.2 ± 6.2	0.441
Extension	75.7 ± 7.9	75.4 ± 5.9	0.445
Flexion	73.6 ± 7.9	69.6 ± 7.8	**0.042**
Pain score	0.2 ± 0.4	0.4 ± 0.7	0.100
Satisfaction	3.6 ± 1.1	2.9 ± 1.2	**0.034**

^*^p-values in bold represent values considered statistically significant (*p* < 0.05)

^a^Group A: patients who underwent intramedullary nailing

^b^Group B: patients who underwent plating osteosynthesis

^c^*MMWS* Modified Mayo Wrist Score

### Radiographic analysis

Radiographic parameters (radial height, radial inclination, and volar tilt) were compared between the 2 groups in terms of preoperative alignment, postoperative alignment after 2 years follow-up, and correction of deformity (Table 3). All 3 preoperative parameters including radial height, radial inclination and volar tilt, postoperative radial volar tilt, and correction of all the 3 parameters showed no significant difference. Only postoperative radial height and radial inclination exhibited significant difference between groups A and B.

**Table 3:** Radiographic analysis

Items	Group A^a^ (*N* = 22)	Group B^b^ (*N* = 25)	*P value* ^*^
Radial height (mm)			
Preoperative	8.4 ± 2.8	7.2 ± 2.5	0.064
Postoperative^c^	11.6 ± 2.6	10.2 ± 3	**0.039**
Correction^d^	3.3 ± 2.8	3 ± 2.1	0.357
Radial inclination (°)			
Preoperative	15.3 ± 5.6	12.7 ± 4	0.067
Postoperative	20.8 ± 2.8	17.7 ± 4.1	**0.004**
Correction	5.6 ± 5.1	5.1 ± 2.7	0.351
Volar tilt (°)			
Preoperative	−6 ± 9.5	−3.7 ± 10.3	0.474
Postoperative	3.8 ± 4.9	2.2 ± 3.3	0.215
Correction	9.7 ± 8.9	8.3 ± 7.2	0.547

^*^P-values in bold are considered statistically significant (*p* < 0.05)

^a^Group A: patients who underwent intramedullary nailing

^b^Group B: patients who underwent plating osteosynthesis

^c^Postoperative 2-year

^d^Deformity correction = postoperative value minus preoperative value of the evaluated parameter

### Complications

There were no major complications such as vascular injury, deep infection, nonunion or tendon rupture. Minor complications were found in 5 patients of group A (23%) and in 10 patients of group B (40%) and included sensory nerve injury, superficial infection, screw migration, and hardware removal due to irritation symptoms (Table 4). No significant differences were noted between the 2 groups regarding over complication rate, superficial infection rate, and incidence of paresthesia. Four patients in group A experienced transient paresthesia along the radial side of the hand, which completely resolved with follow-up. Two patients in group B complained of paresthesia over the palm; 1 patient completely recovered while the other did not. Superficial infection was noted in 2 patients of group B; both resolved after oral antibiotic treatment. Significant difference was noted in implant-related complications including screw migration and hardware irritation. Subsequent removal of plate and screw were performed in 9 patients of group B.

**Table 4:** Surgical complications

Items	Group A^a^	Group B^b^	*P-value* ^*^
Patients with complications	5 (23%)	10 (40%)	0.205
Paresthesia	4 (18%)	2 (8%)	0.297
Superficial infection	0	2 (8%)	0.161
Implant related	2 (9%)	9 (36%)	**0.030**
Migration	2 (9%)	3 (12%)	
Removal	0 (0%)	6 (24%)	

^*^P-values in bold are considered statistically significant (*p* < 0.05)

^a^Group A: patients who underwent intramedullary nailing

^b^Group B: patients who underwent plating osteosynthesis

## Discussion

Malalignment following distal radius fractures may result in altered wrist kinematics [4, 5] and midcarpal instability [15] with reduced motion range and grip strength [16, 17] and eventually lead to unsatisfactory outcomes. Given that the axial shortening of the radius affects treatment outcome in healed extra-articular fractures, the loss of radial length should be a critical predictor [18]. Therefore, the restoration of the anatomical alignment is the primary goal in treatment of posttraumatic deformity of the distal radius and rigid fixation is crucial to prevent secondary collapse and displacement. Fundamentally, the aim of the extra-articular osteotomy is to restore the volar tilt in the sagittal plane, radial inclination in the frontal plane and radial length [13, 19]. Various techniques have been described [20–22]. Open wedge osteotomy has been considered the preferable technique and can be performed in either a dorsal or palmar approach; however, the dorsal approach may compromise wrist flexion due to scarring and implant irritation [23]. In our study, better wrist flexion and patient satisfaction were achieved in the intramedullary nail group (group A) using dorsal open wedge osteotomy and intramedullary fixation. While favorable results could be attributed to more effective osteotomy and less invasive surgical approach used in the nail group, larger sample size and control studies would be necessary to clarify the clinical relevance and functional impact of patient satisfaction and radiographic outcome.

Osteosynthesis with dorsal and/or volar plates allows accurate reduction and secure fixation by sufficient exposure and direct visualization. Criticisms have been raised regarding wide tissue dissection and tendon attrition with internal fixation plate [24]. In our series, as many as 24% of cases in the plate group requested subsequent implant removal. The design of juxta-articular seating could be a potential weakness; however, this was the only locking plate available when we started the treatment protocol, and still commonly adopted currently because of simplicity in application. Implant-related soft tissue irritation was uncommon in our experience. A significantly higher incidence of implant-related complications may, at least partially account for the lower satisfaction in the plate group. Surgical complications of intramedullary nail fixation may include dorsal superficial radial sensory nerve injury and potentially suboptimal reduction due to inadequate surgical exposure [25]. Higher percentage of transient paresthesia in the nail group should caution the surgeon to aim for a more meticulous soft tissue and to protect the nerve during surgical dissection.

While most authors agreed that intramedullary nails yielded comparable surgical outcomes in acute fractures of the distal radius [23–30], only a few of studies indicated the application of intramedullary nails in malunion [31, 32]. In 2 previous studies, the technique and effectiveness of an intramedullary nail combined with bone graft or graft substitute has been demonstrated for the correction of extra-articular malunions of the distal radius. However, there has been no comparison study for the 2 implants in distal radius malunions. Both plate and nail groups in our study exhibited encouraging functional outcomes. Radiographic analysis confirmed the efficacy in correction of all 3 parameters with a significantly better postoperative radial height and inclination in the intramedullary nail group. Although there has been no clear correlation between radiographic findings and functional end-results [33], the values of the postoperative radiographic parameters achieved may be indicative of the quality of surgical realignment after corrective osteotomy. Since open wedge osteotomy instead of distraction osteotomy was performed in all our cases, we did not intentionally correct ulnar variance by lengthening the osteotomy gap. Therefore, our radiographic assessment did not include ulnar variance.

Several studies comparing intramedullary nail with a locking plate in the treatment of distal radius fractures are currently available in the literature [28, 30, 34, 35]. All studies concluded that both devices achieved equally good functional outcomes; while a systemic review indicated that intramedullary nails facilitated favorable functional results with radiological and clinical parameters nearly equivalent to locking plates [36]. These studies all focused on acute fractures; there has so far been no comparison study addressing distal radius malunion. We used eligibility criteria similar to those in a previous study [32] to include all cases with nascent malunion of 4 weeks and more. The surgical technique used in deformity correction in nascent malunion was similar to that used in solid malunion, but could be achieved only through a mini-open incision [12, 32]. A locally resected exuberant callus was used to fill the osteotomy gap in all cases. Recent studies have indicated there are no advantages from cortical and cancellous autografts in terms of radiographic and function results, provided sufficient reduction and stable fixation is achieved [37, 38]. Nevertheless, more complex surgery would be necessary for complicated malunion with severe deformity and soft tissue contracture.

Our study encompasses all the limitations of a retrospective design by relying on medical records and operative notes for data collection. The surgical approach and fixation modality were empirically determined through the planning and experience of a single surgeon. The cases were not randomized and lacked a control group. Patient characteristics varied between two groups including age, sex, and dominant hand involvement; all those could be factors bias affecting the results while no significant difference. Despite being a cohort study including two surgical groups, the technique of open wedge osteotomy differed between the 2 groups as the use of an intramedullary implant required only soft tissue dissection limited to the exposed metaphysis.

## Conclusion

Open-wedge osteotomy with either intramedullary nail or locking plate fixation is a feasible approach for deformity correction in late-diagnosed distal radius fractures. In the present study, both techniques yielded comparable functional and radiographic outcomes. However, the intramedullary nail seemed to facilitate restoration of radial height and inclination with better wrist flexion, less implant-related complications, and higher patient satisfaction. Considering the relative benefits of the surgical procedure, we recommend that both surgical modalities should be applied in nascent and solid malunions. Intraoperative nerve injury was found to be a critical concern, and surgeons should be cautioned when selecting suitable candidates and surgical approaches.

## Abbreviations

MMWS: Modified Mayo Wrist Score
VAS: Visual analog scale
